# Allostatic Load, Depressive Symptoms, and Risk of New-Onset Arthritis Among Middle-Aged and Older Adults: A Prospective Study Based on CHARLS

**DOI:** 10.3390/healthcare14203392

**Published:** 2026-10-12

**Authors:** Ru Feng, Min Ni, Xiaoxin Wang, Guina Tan, Feng Gao, Degang Yang, Jianjun Li

**Affiliations:** 1 School of Rehabilitation, Capital Medical University, Beijing 100068, China; 2 Department of Spinal and Neural Functional Reconstruction, China Rehabilitation Research Center, Beijing 100068, China; 3 Institute of Rehabilitation Medicine, China Rehabilitation Research Center, Beijing 100068, China; 4 School of Rehabilitation, Gannan Medical University, Ganzhou 341000, China; 5 Shenzhen University General Hospital, Shenzhen 518055, China; 6 The Second Affiliated Hospital, Zhejiang University School of Medicine, Hangzhou 310009, China

**Keywords:** allostatic load, depressive symptoms, new-onset arthritis, CHARLS, older adults

## Abstract

**Background**: Arthritis is a major cause of pain, functional limitation, and disability among middle-aged and older adults. Although allostatic load (AL) and depressive symptoms have each been linked to adverse health outcomes, prospective evidence on their independent and joint associations with incident arthritis remains limited, particularly in Chinese populations. **Methods**: This prospective cohort study used data from the China Health and Retirement Longitudinal Study (CHARLS). A total of 8882 participants aged 45 years and older without arthritis at baseline in 2011 were included and followed through 2020. AL was calculated from 11 equally weighted baseline biomarkers (body mass index, systolic and diastolic blood pressure, blood urea nitrogen, total cholesterol, high- and low-density lipoprotein cholesterol, C-reactive protein, glycated hemoglobin, cystatin C, and fasting glucose) using prespecified risk thresholds and categorized as low (0–1), medium (2–3), or high (4–11) burden. Depressive symptoms were measured at baseline using the 10-item Center for Epidemiologic Studies Depression Scale (CESD-10), with a cutoff score of 10. New-onset (incident) arthritis was defined as a newly reported physician diagnosis of “arthritis or rheumatism” during follow-up. Cox proportional hazards models were used to estimate hazard ratios (HRs) and 95% confidence intervals (CIs), adjusting for potential confounders. Multiplicative and additive interactions between AL and depressive symptoms were also examined. **Results**: During follow-up, both higher AL and depressive symptoms were independently associated with an increased risk of new-onset arthritis. In the fully adjusted model, compared with the low-AL group, the HRs for incident arthritis were 1.10 (95% CI: 1.02–1.20) for the medium-AL group and 1.21 (95% CI: 1.04–1.35) for the high-AL group. Participants with depressive symptoms had a significantly higher risk of incident arthritis than those without depressive symptoms (HR = 1.53, 95% CI: 1.41–1.65). No significant multiplicative interaction was observed between AL and depressive symptoms (*p* for interaction = 0.192). Additive interaction analysis suggested limited evidence of excess joint risk in participants with high AL and depressive symptoms, with an attributable proportion of 0.19 (95% CI: 0.02–0.37). **Conclusions**: Higher allostatic load and depressive symptoms were independently associated with an elevated risk of new-onset arthritis among middle-aged and older adults. Although no significant multiplicative interaction was identified, their co-occurrence may confer additional risk on an additive scale. These findings highlight the potential relevance of considering both cumulative physiological stress and psychological distress in arthritis risk assessment.

## 1. Introduction

Arthritis and related musculoskeletal conditions are major contributors to pain, functional limitation, and disability worldwide. According to a systematic analysis for the Global Burden of Disease Study in 2021, 595 million people globally had osteoarthritis in 2020, affecting 7.6% of the global population and making it the most common form of arthritis, with a particularly significant impact on populations over 30 years of age, affecting 15% of this demographic [1]. The epidemiological burden of arthritis in China is increasingly prominent in the context of population ageing. A national CHARLS-based study reported that symptomatic knee osteoarthritis affected approximately 8.1% of Chinese middle-aged and older adults, with prevalence increasing with age [2]. Given the rapid population ageing occurring in many countries, identifying modifiable determinants of new-onset arthritis has important clinical and public health significance.

Allostatic load (AL), a concept derived from stress biology, reflects the cumulative “wear and tear” on the body caused by repeated or prolonged activation of adaptive physiological systems [3,4,5]. It is typically operationalized as a multisystem biomarker-based index capturing dysregulation in metabolic, cardiovascular, inflammatory, and neuroendocrine pathways [5,6,7]. From a biological perspective, chronic stress and elevated AL may disrupt immune homeostasis through dysregulation of the hypothalamic–pituitary–adrenal axis and persistent sympathetic activation [8,9]. These stress-related physiological and immune disturbances provide a plausible biological framework linking cumulative stress burden to arthritis development [9,10].

Accumulating evidence suggests that chronic stress may play a role in the development of arthritis, although evidence differs across arthritis types. A scoping review concluded that most identified studies supported a relationship between chronic stress exposure and arthritis development [10]. Earlier work in the Boston Puerto Rican Health Study found that higher AL was associated with self-reported arthritis among several chronic conditions, although arthritis subtypes were not differentiated [11]. With respect to osteoarthritis (OA)**,** a recent population-based study reported cross-sectional associations between higher AL, OA, and joint pain, while emphasizing the need for longitudinal investigation [12]. For rheumatoid arthritis (RA), more recent prospective evidence from the UK Biobank showed that individuals with the highest AL had a higher subsequent risk of RA than those with the lowest AL [9]. In parallel, studies from the United States and the United Kingdom have linked higher AL with chronic pain and greater pain severity, respectively, further supporting the plausibility of AL as a marker of musculoskeletal vulnerability [13,14]. Taken together, these findings support a potential link between cumulative physiological dysregulation and arthritic outcomes, but the available evidence remains heterogeneous with respect to arthritis phenotype, AL construction, population, and study design, and prospective evidence for broadly defined incident arthritis remains limited.

Depressive symptoms (DepS) may represent another important determinant of arthritis onset. For broadly defined arthritis, longitudinal evidence has been relatively consistent. Prior longitudinal studies have shown that depressive symptoms are associated with incident arthritis [15], while a subsequent CHARLS study found that persistent or worsening depressive symptom trajectories were associated with increased risk of new-onset arthritis [16]. More recently, a pooled analysis of six longitudinal ageing cohorts across multiple countries similarly found that depression was independently associated with incident arthritis [17]. For RA, population-based longitudinal studies have also reported associations between depression and subsequent RA. Population-based cohort studies have also linked depression to subsequent rheumatoid arthritis risk [18], while data from the Nurses’ Health Study showed that depression was associated with subsequent RA, particularly seronegative RA [19]. A 2022 meta-analysis further supported a bidirectional association between rheumatoid arthritis and depression, suggesting close interconnections between psychological distress, inflammation, and rheumatic disease [20]. Evidence specifically addressing depression as a predictor of incident OA, however, remains comparatively less established. Collectively, these findings support an association between psychological distress and subsequent arthritis, while also indicating heterogeneity according to arthritis phenotype and study population.

Importantly, AL and depressive symptoms may represent interrelated rather than entirely independent dimensions of stress-related vulnerability. Longitudinal evidence suggests a bidirectional relationship between depressive symptoms and AL, indicating that psychological distress and cumulative physiological dysregulation may influence each other over time [21]. Chronic stress and depression are also closely linked to alterations in neuroendocrine stress responses and inflammatory signaling, providing potential biological pathways shared with the physiological dysregulation captured by AL [22,23]. In rheumatoid arthritis, immune-mediated inflammation has been proposed as a biological link with depression, with overlapping peripheral and central immunological mechanisms implicated in both conditions [24]. Together, these findings provide biological plausibility for considering AL and depressive symptoms within a shared stress–inflammation framework when examining arthritis risk. However, despite growing evidence linking AL and depressive symptoms individually to arthritic outcomes, these two dimensions have largely been investigated separately. Prospective studies simultaneously evaluating cumulative physiological dysregulation and psychological distress in relation to incident arthritis remain limited, and their independent and joint associations have not been well characterized, particularly in middle-aged and older Chinese populations. Clarifying these relationships may improve understanding of arthritis risk and identify subgroups with concurrent physiological and psychological vulnerability.

Therefore, in this nationwide longitudinal cohort of middle-aged and older Chinese adults without arthritis at baseline, we aimed to examine the associations of medium and high versus low allostatic load burden and the presence versus absence of depressive symptoms with the risk of new-onset arthritis, and to further assess their joint and interaction effects. 

## 2. Methods

### 2.1. Study Population and Setting

This study utilizes data from the CHARLS, a nationally representative longitudinal cohort study initiated in 2011 [25]. The CHARLS is an ongoing, nationally representative longitudinal study of adults aged 45 and older in China. Study participants were interviewed beginning in 2011 and followed up every 2 years thereafter, and details on health, economic, and social circumstances were collected. Further details of the study design have been described previously. All participants provided written informed consent. Ethical approval for the study was granted by the Ethics Review Committee of Peking University (IRB00001052-11015). The study was conducted and reported in accordance with the STROBE guidelines.

**Figure 1:**
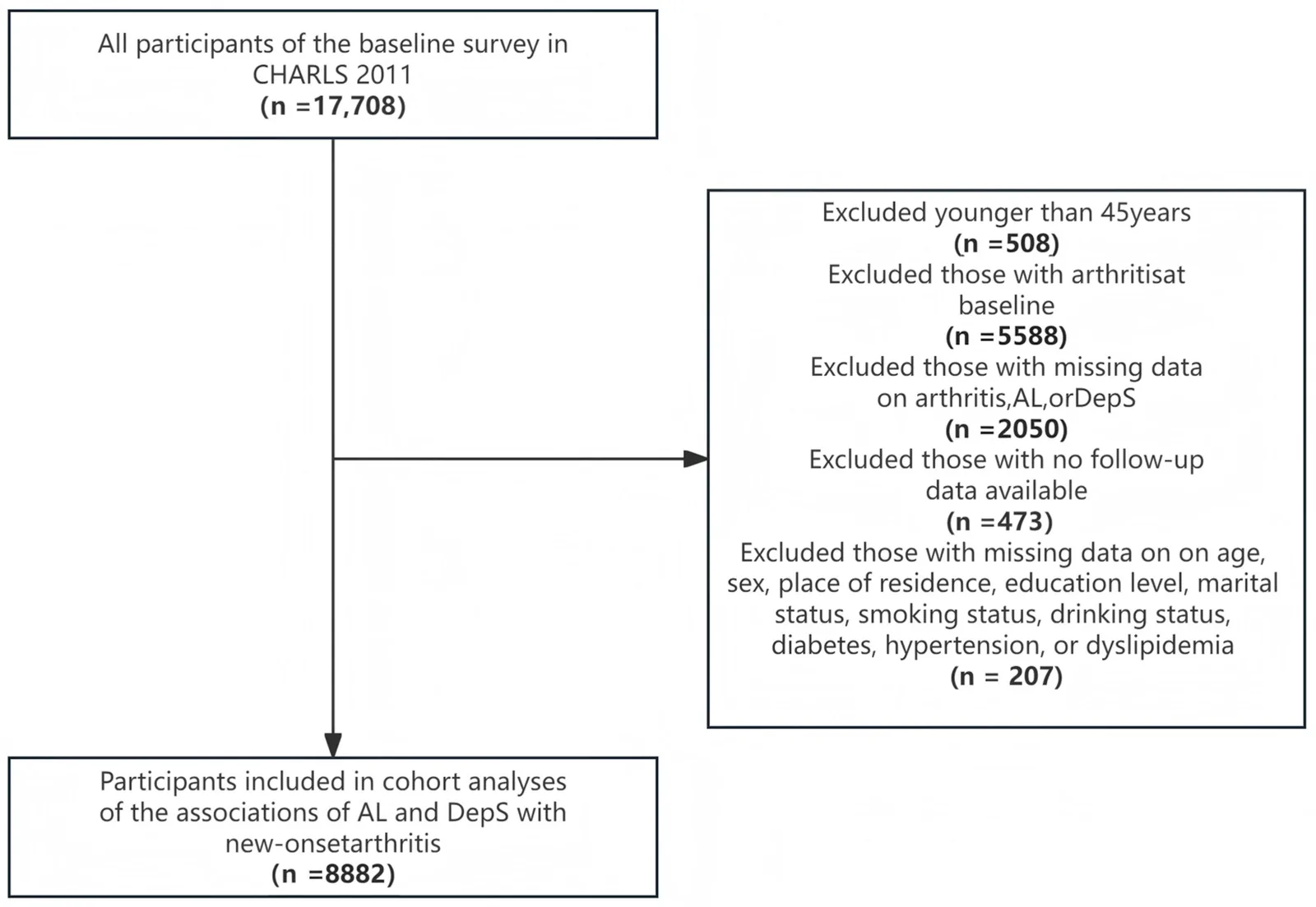
The selection process of study population. Abbreviations: AL, allostatic load; DepS, Depressive Symptoms.

Data from the 2011 baseline survey were analyzed, which initially included 17,708 participants. We excluded participants younger than 45 years (*n* = 508), those with arthritis at baseline (*n* = 5588), those with missing data on arthritis, AL, or DepS (*n* = 2050), those with no follow-up data available (*n* = 473) and those with missing data on age, sex, place of residence, education level, marital status, smoking status, drinking status, diabetes, hypertension, and dyslipidemia (*n* = 207). A total of 8882 participants were ultimately included in the longitudinal analysis. The primary analyses were conducted using a complete-case approach. The detailed flow chart of the inclusion and exclusion process is presented in Figure 1.

### 2.2. Allostatic Load

AL was used to capture cumulative physiological dysregulation across multiple biological systems. It was calculated using baseline biomarkers measured in 2011, including body mass index (BMI), systolic blood pressure (SBP), diastolic blood pressure (DBP), blood urea nitrogen (BUN), total cholesterol (TC), high-density lipoprotein cholesterol (HDL-C), low-density lipoprotein cholesterol (LDL-C), C-reactive protein (CRP), glycated hemoglobin (HbA1c), cystatin C, and fasting blood glucose [26]. These biomarkers represented four physiological systems: (1) the metabolic system (BMI, TC, HDL-C, LDL-C, HbA1c, cystatin C, and fasting blood glucose); (2) the cardiovascular system (SBP and DBP); (3) the renal system (BUN and cystatin C); and (4) the inflammatory system (CRP). High-risk levels for individual biomarkers were defined according to commonly used clinical cutoff points, following the approach used in previous CHARLS-based research [26]; the specific criteria are provided in Supplementary Table S1. For each biomarker, a score of 1 was assigned for values in the high-risk range and 0 otherwise. The total AL score was generated by summing all components, with higher scores indicating a greater physiological burden. Medication use was not incorporated into the AL scoring algorithm. Following previously established categories, AL scores were classified as low (0–1), medium (2–3), and high (4–11) burden [26,27].

### 2.3. Assessment of Depressive Symptoms

Depressive symptoms were assessed at baseline in 2011 using CESD-10. The total score ranged from 0 to 30, with higher scores indicating more severe depressive symptoms [28]. Participants were asked to recall their experiences during the previous week and indicate how often they had experienced each emotion listed on the scale. A cutoff score of 10 was used to classify participants as having or not having depressive symptoms (DepS), as this threshold has been shown to identify individuals with clinically relevant DepS [29].

### 2.4. Assessment of Arthritis

New-onset (incident) arthritis was ascertained using the standardized CHARLS questionnaire. At baseline and each follow-up wave, participants were asked whether they had been diagnosed with “arthritis or rheumatism” by a doctor. Participants who reported no such diagnosis at baseline in 2011 but first reported a physician diagnosis of arthritis or rheumatism during the 2013, 2015, 2018, or 2020 follow-up were classified as having new-onset arthritis.

### 2.5. Covariates

Potential covariates were selected a priori based on clinical relevance and the previous literature, including age, sex, place of residence, education level, marital status, smoking status, drinking status, diabetes, hypertension, and dyslipidemia. All covariates were measured at baseline in 2011. Physical activity was assessed using three binary indicators reflecting progressively more stringent activity criteria: any vigorous-, moderate-, or light-intensity physical activity for at least 10 min per week; moderate-to-vigorous physical activity for at least 10 min per week; and moderate-to-vigorous physical activity on at least 3 days per week for at least 10 min per session. Because physical activity data were substantially incomplete, these variables were included only in sensitivity analyses among participants with complete physical activity data.

### 2.6. Statistical Analysis

Baseline characteristics were described according to allostatic load burden groups. Continuous variables were presented as mean ± standard deviation (SD), and categorical variables were expressed as numbers and percentages. Differences among groups were compared using analysis of variance for continuous variables and the chi-square test for categorical variables.

Cox proportional hazards regression models were used to estimate hazard ratios (HRs) and 95% confidence intervals (CIs) for the associations of allostatic load burden and depressive symptoms with new-onset arthritis. The proportional hazards assumption was assessed using Schoenfeld residuals. Three models were constructed. For the association between allostatic load burden and new-onset arthritis, Model 1 was unadjusted; Model 2 was adjusted for depressive symptoms; and Model 3 was further adjusted for age, sex, place of residence, education level, marital status, smoking status, drinking status, diabetes, hypertension, and dyslipidemia. For the association between depressive symptoms and new-onset arthritis, Model 1 was unadjusted; Model 2 was adjusted for allostatic load burden; and Model 3 was further adjusted for the above covariates. To assess interaction effects between allostatic load burden and depressive symptoms, both multiplicative and additive interactions were examined. Multiplicative interaction was evaluated by including a product term in the Cox regression model. Additive interaction was assessed using the relative excess risk due to interaction (RERI), attributable proportion due to interaction (AP), and synergy index (SI). Given that death may preclude the occurrence of the study outcome, competing-risk sensitivity analyses were performed using Fine–Gray subdistribution hazard models, treating death before incident arthritis as a competing event. To further assess potential confounding by physical activity, an additional sensitivity analysis was conducted among participants with available physical activity data. In this analysis, Model 1 was unadjusted; Model 2 was adjusted for age, sex, place of residence, education level, marital status, smoking status, drinking status, diabetes, hypertension, and dyslipidemia, with depressive symptoms and allostatic load burden additionally adjusted for each other as appropriate; and Model 3 was further adjusted for physical activity. All statistical analyses were conducted using R Studio (Version 4.4.2) and Zstats v1.0 (www.zstats.net). A two-sided *p* value < 0.05 was considered statistically significant.

## 3. Results

### 3.1. Baseline Characteristics

A total of 8882 participants were included in the analysis, of whom 4890, 2971, and 1021 were classified into the low-, medium-, and high-burden groups, respectively, according to allostatic load burden. The median follow-up time was 8.9 years (IQR, 4.1–9.0 years). During follow-up, 974 participants died. The mean age of the overall population was 58.74 ± 9.46 years, and participants in the higher-burden groups were older than those in the lower-burden group (57.27 ± 8.93, 60.10 ± 9.69, and 61.84 ± 9.92 years for the low-, medium-, and high-burden groups, respectively; *p* < 0.001).

Significant between-group differences were observed in sex, place of residence, marital status, educational level, smoking status, drinking status, diabetes, hypertension, dyslipidemia, and physical activity (all *p* < 0.05). Compared with participants in the low-burden group, those in the high-burden group tended to be older and had a higher prevalence of cardiometabolic comorbidities. No significant difference in depressive symptoms was found across the three burden groups (*p* = 0.747). As expected, the distributions of individual allostatic load components varied across AL burden groups, with participants in the high-burden group generally showing less favorable metabolic, cardiovascular, and inflammatory profiles (Table 1).

### 3.2. Association Between Allostatic Load Burden and New-Onset Arthritis

We next examined the independent association between allostatic load burden and the risk of new-onset arthritis using Cox regression models (Table 2). In the crude model, compared with the low-burden group, both the medium- and high-burden groups had significantly increased risks of new-onset arthritis, with HRs of 1.10 (95% CI: 1.01–1.19, *p* = 0.025) and 1.16 (95% CI: 1.03–1.32, *p* = 0.011), respectively. After adjustment for depressive symptoms, the associations remained statistically significant, with HRs of 1.11 (95% CI: 1.02–1.20, *p* = 0.015) for the medium-burden group and 1.18 (95% CI: 1.04–1.33, *p* = 0.009) for the high-burden group.

In the fully adjusted model, which additionally controlled for age, sex, place of residence, drinking status, diabetes, hypertension, dyslipidemia, education level, marital status, smoking status, and depressive symptoms, elevated allostatic load burden remained independently associated with a higher risk of new-onset arthritis. Compared with the low-burden group, the adjusted HRs were 1.10 (95% CI: 1.02–1.22, *p* = 0.028) for the medium-burden group and 1.21 (95% CI: 1.04–1.35, *p* = 0.006) for the high-burden group.

### 3.3. Association Between Depressive Symptoms and New-Onset Arthritis

We then assessed whether depressive symptoms were independently associated with the risk of new-onset arthritis using Cox regression models (Table 3). In the crude model, participants with depressive symptoms had a significantly higher risk of new-onset arthritis than those without depressive symptoms (HR = 1.65, 95% CI: 1.53–1.78, *p* < 0.001). After adjustment for allostatic load burden, the association remained significant (HR = 1.66, 95% CI: 1.53–1.79, *p* < 0.001).

In the fully adjusted model, which further controlled for age, sex, place of residence, drinking status, diabetes, hypertension, dyslipidemia, education level, marital status, smoking status, and allostatic load burden, depressive symptoms remained independently associated with an increased risk of new-onset arthritis (HR = 1.53, 95% CI: 1.41–1.65, *p* < 0.001).

### 3.4. Joint Association and Interaction Between Allostatic Load Burden and Depressive Symptoms on New-Onset Arthritis

We further examined the joint associations of allostatic load burden and depressive symptoms with new-onset arthritis and assessed their interactions on multiplicative and additive scales (Table 4 and Table 5). Using participants with low allostatic load burden and no depressive symptoms as the common reference group, the adjusted HRs (95% CIs) were 1.44 (1.29–1.60) for low burden with depressive symptoms, 1.05 (0.94–1.17) for medium burden without depressive symptoms, 1.69 (1.50–1.92) for medium burden with depressive symptoms, 1.10 (0.93–1.29) for high burden without depressive symptoms, and 1.92 (1.58–2.32) for high burden with depressive symptoms. No statistically significant multiplicative interaction was observed between allostatic load burden and depressive symptoms (*p* for interaction = 0.192).

Additive interaction was further evaluated using the relative excess risk due to interaction (RERI), attributable proportion due to interaction (AP), and synergy index (SI). In the medium-burden group, the estimates for additive interaction were not statistically significant, with RERI of 0.20 (95% CI: −0.03 to 0.43), AP of 0.12 (95% CI: −0.01 to 0.25), and SI of 1.42 (95% CI: 0.91 to 2.20). In the high-burden group, the AP was statistically significant [0.19 (95% CI: 0.02 to 0.37)], suggesting that approximately 19% of the excess risk among participants with both high allostatic load burden and depressive symptoms might be attributable to their interaction on an additive scale. However, the corresponding RERI [0.37 (95% CI: −0.02 to 0.76)] and SI [1.69 (95% CI: 0.98 to 2.90)] did not reach statistical significance (Table 5). Overall, these findings suggest no significant multiplicative interaction and only limited evidence for additive interaction between allostatic load burden and depressive symptoms.

### 3.5. Sensitivity Analyses

In Fine–Gray competing-risk analyses treating death before the occurrence of new-onset arthritis as a competing event, the fully adjusted sHRs were 1.08 (95% CI, 1.00–1.18; *p* = 0.064) for medium allostatic load burden and 1.11 (95% CI, 0.98–1.26; *p* = 0.099) for high allostatic load burden, compared with low allostatic load burden. Depressive symptoms remained significantly associated with new-onset arthritis after accounting for competing mortality (sHR, 1.50; 95% CI, 1.38–1.62; *p* < 0.001) (Supplementary Tables S2 and S3).

In sensitivity analyses restricted to participants with available physical activity data, additional adjustment for the three physical activity indicators had little influence on the effect estimates. The association between depressive symptoms and incident arthritis remained statistically significant (HR, 1.60; 95% CI, 1.43–1.81), whereas the associations for medium and high AL burden did not reach statistical significance (HR, 1.03; 95% CI, 0.91–1.17 and HR, 1.19; 95% CI, 0.98–1.44, respectively) (Supplementary Table S4).

The proportional hazards assumption was assessed for all Cox regression analyses, with no evidence of violation based on Schoenfeld residuals.

## 4. Discussion

In this prospective cohort study, we found that higher allostatic load burden and depressive symptoms were each independently associated with an increased risk of new-onset arthritis. In addition, although no significant multiplicative interaction was observed, additive interaction analysis suggested limited evidence of excess joint risk among participants with a high allostatic load burden. In sensitivity analyses restricted to participants with available physical activity data, additional adjustment for physical activity had little influence on the effect estimates. The association with depressive symptoms remained statistically significant, whereas the associations with AL did not reach statistical significance in the substantially reduced sample resulting from missing physical activity data. The association between AL and incident arthritis was attenuated in the competing-risk analysis and no longer reached statistical significance, although the direction of association remained unchanged. This attenuation may partly reflect the competing influence of mortality in this older population, as individuals with greater physiological burden may die before arthritis is observed, thereby reducing the cumulative incidence of the study outcome. In contrast, the association with depressive symptoms remained evident after accounting for competing mortality, suggesting greater consistency of this association across the two analytical approaches. Taken together, these findings suggest that the broad CHARLS-defined arthritis outcome was associated with both cumulative physiological burden and psychological distress.

A key contribution of this study is that it places incident arthritis within an allostatic framework. Allostatic load was originally proposed to capture the cumulative biological “wear and tear” caused by chronic stress across multiple physiological systems [4]. The biomarkers commonly used to construct allostatic load reflect metabolic, cardiovascular, and inflammatory domains that may also be relevant to arthritic outcomes [13]. Accordingly, the association of cumulative physiological dysregulation with arthritis may vary across subtypes according to their distinct pathophysiological mechanisms and the relative contributions of inflammatory and mechanical processes.

Although direct evidence linking allostatic load to arthritis remains relatively limited, findings have been broadly consistent. In the Americas, Mattei et al. (2010) reported a significant dose–response between AL and self-reported arthritis in Boston Puerto Rican adults; participants with an AL score ≥ 6 had more than twice the odds of self-reported arthritis compared with those with an AL score of 0–1 (OR = 2.17, 95% CI: 1.25–3.67) [11]. In Australia, Schwetlik et al. subsequently examined 1600 participants from the North West Adelaide Health Study and reported that each one-unit increase in allostatic load was associated with 12% higher adjusted odds of osteoarthritis and 11% higher odds of joint pain [12]. More recently, prospective evidence from Europe has emerged from the UK Biobank. Among 186,310 participants, those in the highest allostatic load quartile had a higher subsequent risk of rheumatoid arthritis than those in the lowest quartile (HR = 1.52) [9]. These studies provide relevant context but are not directly comparable because they differ substantially in AL construction, arthritis phenotype, and study design. The comparatively modest effect estimate observed in our study (HR = 1.21 for high versus low allostatic load) may partly reflect differences in biomarker composition, population characteristics, and the broader arthritis outcome used in CHARLS.

The association between depressive symptoms and subsequent arthritis is supported by a larger and geographically diverse body of evidence. In Asia, using CHARLS, Ke et al. (2021) reported that baseline depression was associated with increased odds of incident arthritis during follow-up (adjusted OR = 1.56, 95% CI 1.37–1.79) compared with no baseline depression [15]. Subsequent Chinese studies further demonstrated the importance of the longitudinal pattern of depressive symptoms. He et al. used CHARLS (*n* = 5583, 2011–2018), identified five depressive symptom trajectories and, versus the stable-low group, reported adjusted HRs for incident arthritis of 1.64 (95% CI 1.30–2.07) for stable-moderate and 2.19 (95% CI 1.38–3.48) for stable-high, with increasing and decreasing trajectories also elevated (HR 1.86 and 1.99, respectively) [16].

Comparable findings have been reported outside Asia. Using UK primary-care records, Vallerand et al. compared 403,932 individuals with major depressive disorder with more than 5.3 million individuals without depression and found a 38% higher adjusted risk of incident rheumatoid arthritis among those with major depressive disorder [18]. In the United States, pooled data from 195,358 women in the Nurses’ Health Study and Nurses’ Health Study II showed that depression was associated with subsequent rheumatoid arthritis (HR = 1.28), with a stronger association for seronegative rheumatoid arthritis (HR = 1.63) [19]. European evidence also supports a broader role for psychosocial wellbeing in arthritis development: in the Survey of Health, Ageing and Retirement in Europe, higher subjective wellbeing predicted a lower risk of incident arthritis during nine years of follow-up [30]. Most importantly, a pooled analysis of six ageing cohorts (HRS, MHAS, SHARE, ELSA, KLoSA, CHARLS; *n* = 68,445 adults ≥ 50 y from the US, Mexico, continental Europe, England, Republic of Korea, and China) by Duan et al. (2025) found depression to be independently associated with incident arthritis (adjusted HR = 1.37, 95% CI 1.32–1.42), alongside low SES (HR = 1.36), over a median 5.9-year follow-up [17]. Our estimate for depressive symptoms (HR = 1.53) was broadly consistent with previous longitudinal findings, although differences in arthritis definitions should be considered when comparing effect estimates.

The relationship may also be bidirectional. In addition to evidence that depression precedes arthritis, longitudinal studies have shown that osteoarthritis and rheumatoid arthritis are associated with an increased subsequent risk of depressive symptoms or clinically diagnosed depression [20,31,32,33]. This bidirectionality suggests that psychological distress should not be regarded as solely because of pain and disability after arthritis develops. Instead, depressive symptoms may be involved earlier in the disease trajectory, while established arthritis may in turn aggravate psychological distress through pain, functional limitation, inflammation, and reduced social participation. Potential pathways linking depression and arthritis include dysregulation of stress-response systems, altered immune and inflammatory signaling, and adverse behavioral pathways [23,24].

Our interaction findings should be interpreted within this broader stress–psychological framework. Previous studies suggest that depressive symptoms and allostatic load are related rather than completely independent constructs. For example, longitudinal evidence from Puerto Rican older adults has demonstrated a bidirectional association between depressive symptoms and allostatic load [21]. Consistent longitudinal associations between AL and depressive symptoms have also been reported in the English Longitudinal Study of Ageing and the US HANDLS study [34,35]. The six-cohort international study by Duan et al. demonstrated joint effects of depression and socioeconomic disadvantages on arthritis incidence across multiple geographic regions [17]. Nevertheless, to our knowledge, previous population-based prospective studies have not directly evaluated both allostatic load and depressive symptoms, including their additive and multiplicative interaction, in relation to incident arthritis.

In the present study, no statistically significant multiplicative interaction was identified, indicating that the relative association of depressive symptoms with arthritis did not differ clearly according to allostatic load burden. On the additive scale, however, the attributable proportion in the high-allostatic-load group was 0.19, suggesting possible excess risk when high physiological burden and depressive symptoms co-occurred. Thus, rather than establishing biological synergy, our results provide hypothesis-generating evidence that individuals simultaneously experiencing substantial multisystem physiological dysregulation and psychological distress may constitute a particularly vulnerable subgroup.

These findings may have practical implications. First, they support a broader risk-stratification framework for arthritis that incorporates not only conventional demographic and metabolic factors, but also multisystem physiological stress burden and mental health status. Second, depressive symptoms in middle-aged and older adults may have prognostic relevance beyond psychiatric outcomes alone. Third, if confirmed in future studies, the additive interaction signal suggests that individuals with both high allostatic load and depressive symptoms may represent a subgroup for whom early prevention and integrated management could be particularly valuable.

This study has several strengths, including its prospective design, large population-based sample, and simultaneous evaluation of both independent and interaction effects. However, several limitations should be acknowledged. First, causality cannot be inferred because of the observational design. Second, arthritis was based on participants’ reports of a physician diagnosis of “arthritis or rheumatism” rather than independently adjudicated medical records. This broad outcome definition does not distinguish among specific arthritis subtypes, such as osteoarthritis, rheumatoid arthritis, or gout, and may be subject to reporting or misclassification bias. Given the substantial etiological and pathophysiological heterogeneity among these conditions, our findings should therefore be interpreted as relating to the broad CHARLS-defined arthritis outcome and should not be generalized to any specific arthritis subtype. Third, allostatic load is a composite construct, and the use of different biomarker panels or cutoffs may yield different estimates across studies. In addition, AL and depressive symptoms were assessed only at baseline, and changes in these exposures during follow-up were not captured. Thus, the observed associations reflect baseline exposure status and may not account for time-varying physiological or psychological changes. Fourth, physical activity was not included in the primary models because of substantial missing data, although additional adjustment for physical activity in sensitivity analyses resulted in little change in the effect estimates. Residual confounding from other unmeasured lifestyle factors, including diet, cannot be excluded. Finally, attrition and missing data may have introduced selection bias and affected the representativeness of the analytical cohort.

## 5. Conclusions

In conclusion, higher baseline allostatic load and depressive symptoms were associated with an increased risk of new-onset arthritis among middle-aged and older Chinese adults. The association with depressive symptoms persisted after accounting for death as a competing event, whereas that with allostatic load was attenuated. Although no multiplicative interaction was observed, additive interaction analysis suggested a possible excess risk associated with the coexistence of high allostatic load and depressive symptoms. These findings suggest that cumulative physiological dysregulation and psychological distress may represent complementary dimensions of vulnerability associated with subsequent arthritis risk. Further longitudinal studies incorporating repeated assessments and more specific ascertainment of arthritis phenotypes are warranted to validate these findings.

## Figures and Tables

**Table 1:** Baseline characteristics of participants by allostatic load burden.

Variables	Total (*n* = 8882)	Low Burden (*n* = 4890)	Medium Burden (*n* = 2971)	High Burden (*n* = 1021)	*p*
Age	58.74 ± 9.46	57.27 ± 8.93	60.10 ± 9.69	61.84 ± 9.92	<0.001
Sex					0.006
Female	4360 (49.09)	2465 (50.41)	1388 (46.72)	507 (49.66)	
Male	4522 (50.91)	2425 (49.59)	1583 (53.28)	514 (50.34)	
Place of residence					<0.001
Urban	3479 (39.17)	1860 (38.04)	1165 (39.21)	454 (44.47)	
Rural	5403 (60.83)	3030 (61.96)	1806 (60.79)	567 (55.53)	
Marital status					<0.001
Married	7915 (89.11)	4416 (90.31)	2610 (87.85)	889 (87.07)	
Divorced	70 (0.79)	44 (0.90)	21 (0.71)	5 (0.49)	
Separated	823 (9.27)	386 (7.89)	317 (10.67)	120 (11.75)	
Never married	74 (0.83)	44 (0.90)	23 (0.77)	7 (0.69)	
Education level					<0.001
Junior high school and below	5893 (66.35)	3161 (64.64)	2015 (67.82)	717 (70.23)	
Junior high school	1842 (20.74)	1057 (21.62)	582 (19.59)	203 (19.88)	
Senior high school	972 (10.94)	563 (11.51)	329 (11.07)	80 (7.84)	
University or above	175 (1.97)	109 (2.23)	45 (1.51)	21 (2.06)	
Smoking status					<0.001
Never	5312 (58.46)	3005 (59.92)	1713 (56.61)	594 (56.68)	
Previous	821 (9.04)	368 (7.34)	326 (10.77)	127 (12.12)	
Current	2953 (32.50)	1640 (32.70)	986 (32.58)	327 (31.20)	
Drinking status					<0.001
Never	5186 (58.39)	2929 (59.90)	1678 (56.48)	579 (56.71)	
Previous	805 (9.06)	359 (7.34)	321 (10.80)	125 (12.24)	
Current	2891 (32.55)	1602 (32.76)	972 (32.72)	317 (31.05)	
Diabetes					<0.001
No	8356 (94.08)	4719 (96.50)	2785 (93.74)	852 (83.45)	
Yes	526 (5.92)	171 (3.50)	186 (6.26)	169 (16.55)	
hypertension					<0.001
No	6759 (76.10)	4125 (84.36)	2081 (70.04)	553 (54.16)	
Yes	2123 (23.90)	765 (15.64)	890 (29.96)	468 (45.84)	
Dyslipidemia					<0.001
No	8064 (90.79)	4550 (93.05)	2675 (90.04)	839 (82.17)	
Yes	818 (9.21)	340 (6.95)	296 (9.96)	182 (17.83)	
Any PA ≥ 10 min/week					<0.001
No	399 (4.49)	179 (3.66)	160 (5.39)	60 (5.88)	
Yes	3383 (38.09)	1893 (38.71)	1126 (37.90)	364 (35.65)	
Miss	5100 (57.42)	2818 (57.63)	1685 (56.71)	597 (58.47)	
Moderate-to-vigorous PA ≥ 10 min/week					<0.001
No	1324 (14.91)	631 (12.90)	502 (16.90)	191 (18.71)	
Yes	2457 (27.66)	1441 (29.47)	784 (26.39)	232 (22.72)	
Miss	5101 (57.43)	2818 (57.63)	1685 (56.71)	598 (58.57)	
Moderate-to-vigorous PA ≥ 3 days/week, ≥10 min/session					<0.001
No	1464 (16.48)	705 (14.42)	554 (18.65)	205 (20.08)	
Yes	2317 (26.09)	1367 (27.96)	732 (24.64)	218 (21.35)	
Miss	5101 (57.43)	2818 (57.63)	1685 (56.71)	598 (58.57)	
Depressive symptoms					0.747
No	6145 (69.18)	3375 (69.02)	2070 (69.67)	700 (68.56)	
Yes	2737 (30.82)	1515 (30.98)	901 (30.33)	321 (31.44)	
Allostatic load components					
BMI (kg/m^2^)	23.37 ± 3.63	22.59 ± 2.97	23.86 ± 3.98	25.42 ± 4.17	
DBP (mmHg)	75.67 ± 12.21	71.78 ± 9.51	78.91 ± 13.28	83.90 ± 13.00	
SBP (mmHg)	129.40 ± 21.24	121.46 ± 15.80	135.81 ± 22.70	146.85 ± 21.70	
Bun (mg/dL)	15.69 ± 4.43	14.83 ± 3.56	16.08 ± 4.76	17.20 ± 5.24	
TC (mg/dL)	192.65 ± 38.28	183.61 ± 28.60	194.06 ± 38.76	215.40 ± 50.00	
HDL_C	50.77 ± 15.34	54.30 ± 13.82	49.38 ± 15.92	43.97 ± 15.23	
LDL_C	115.99 ± 34.99	109.69 ± 25.74	117.38 ± 36.15	130.94 ± 48.19	
CRP (mg/L)	2.52 ± 6.86	1.35 ± 4.14	3.03 ± 8.50	4.63 ± 7.88	
Glycated hemoglobin (%)	5.25 ± 0.83	5.07 ± 0.41	5.25 ± 0.80	5.81 ± 1.40	
Cystatin C (mg/L)	1.01 ± 0.27	0.93 ± 0.17	1.04 ± 0.25	1.13 ± 0.42	
Fasting blood glucose (mg/dL)	109.62 ± 33.80	100.55 ± 13.03	110.33 ± 32.95	134.70 ± 57.47	

*p* values for physical activity were calculated among participants with available physical activity data, excluding missing values.

**Table 2:** Association between allostatic load burden and risk of new-onset arthritis.

Variables	Model1		Model2		Model3	
	HR (95%CI)	*p*	HR (95%CI)	*p*	HR (95%CI)	*p*
AL						
Low burden	1.00 (Reference)		1.00 (Reference)		1.00 (Reference)	
Medium burden	1.10 (1.01~1.19)	0.025	1.11 (1.02~1.20)	0.015	1.10 (1.02~1.20)	0.028
High burden	1.16 (1.03~1.32)	0.011	1.18 (1.04~1.33)	0.009	1.21 (1.04~1.35)	0.006

HR: Hazard Ratio, CI: Confidence Interval. Model1: Crude. Model2: Adjust Depressive symptoms. Model3: Adjust Depressive symptoms, age, sex, place of residence, education level, marital status, smoking status, drinking status, diabetes, hypertension, and dyslipidemia.

**Table 3:** Association between depressive symptoms and risk of new-onset arthritis.

Variables	Model1		Model2		Model3	
	HR (95%CI)	*p*	HR (95%CI)	*p*	HR (95%CI)	*p*
Depressive symptoms						
No	1.00 (Reference)		1.00 (Reference)		1.00 (Reference)	
Yes	1.65 (1.53~1.78)	<0.001	1.66 (1.53~1.79)	<0.001	1.53 (1.41~1.65)	<0.001

HR: Hazard Ratio, CI: Confidence Interval. Model1: Crude. Model2: Adjust Allostatic load. Model3: Adjust Allostatic load, age, sex, place of residence, education level, marital status, smoking status, drinking status, diabetes, hypertension, and dyslipidemia.

**Table 4:** Joint associations of allostatic load burden and depressive symptoms with the risk of new-onset arthritis.

Variables	Case	HR (95%CI)	*p*
AL-low burden			
no	877/3375	1.00 (Reference)	
yes	571/1515	1.44 (1.29~1.60)	<0.001
AL-medium burden			
no	552/2070	1.05 (0.94~1.17)	0.371
yes	374/901	1.69 (1.50~1.92)	<0.001
AL-high burden			
no	187/700	1.10 (0.93~1.29)	0.257
yes	131/321	1.92 (1.58~2.32)	<0.001
*p* for multiplicative interaction = 0.192			

HR: Hazard Ratio, CI: Confidence Interval. Participants with low AL burden and without depressive symptoms served as the common reference group for all joint exposure categories. Adjust: age, sex, place of residence, education level, marital status, smoking status, drinking status, diabetes, hypertension, and dyslipidemia.

**Table 5:** Additive interaction between allostatic load burden and depressive symptoms in relation to new-onset arthritis.

	RERI (95%CI)	AP (95%CI)	SI (95%CI)
AL-medium burden × Deps	0.20 (−0.03~0.43)	0.12 (−0.01~0.25)	1.42 (0.91~2.20)
AL-high burden × Deps	0.37 (−0.02~0.76)	0.19 (0.02~0.37)	1.69 (0.98~2.90)

RERI: relative excess risk due to interaction, AP: attributable proportion, SI: synergy index, CI: Confidence Interval. Additive interaction measures were calculated from the joint hazard ratios presented in Table 4, with participants with low AL burden and without depressive symptoms serving as the common reference group. Adjust: age, sex, place of residence, education level, marital status, smoking status, drinking status, diabetes, hypertension, and dyslipidemia.

## Data Availability

All data can be obtained from https://charls.pku.edu.cn/, and it is publicly available.

## Supplementary Materials

The following supporting information can be downloaded at: https://www.mdpi.com/article/10.3390/healthcare14203392/s1, Table S1: Biomarkers, physiological systems, and high-risk criteria used to construct the allostatic load index; Table S2: Competing risk analysis of the association between allostatic load burden and new-onset arthritis; Table S3: Competing risk analysis of the association between depressive symptoms and new-onset arthritis; Table S4 Sensitivity analyses of the associations of allostatic load and depressive symptoms with new-onset arthritis after additional adjustment for physical activity (*n* = 3780).
