# Supplementary material for: Allostatic Load, Depressive Symptoms, and Risk of New-Onset Arthritis Among Middle-Aged and Older Adults: A Prospective Study Based on CHARLS
Source: Healthcare (Basel). 2026 Oct 12;14(20):3392. doi: 10.3390/healthcare14203392 (PMC13704000; doi:10.3390/healthcare14203392)
Supplement: Supplementary file 1 [file healthcare-14-03392-s001.zip › healthcare-4530747-supplementary.pdf]

Supplementary Table S1. Biomarkers, physiological systems, and high-risk criteria used to construct the allostatic load index

Biomarkers	Cut points
**Metabolic System**	
Body mass index (BMI)	≤18.5 kg/m2; ≥25.0 kg/m2
High-density lipoprotein (HDL) cholesterol	<40 mg/dL
Low-density lipoprotein (LDL) cholesterol	>160 mg/dL
Total cholesterol (TC)	≥240 mg/dL
Glycated hemoglobin (HbA1c)	≥6.5%
Glucose	≥126 mg/dL
**Cardiovascular System**	
Systolic blood pressure (SBP)	≥140 mmHg
Diastolic blood pressure (DBP)	≥90 mmHg
**Renal System**	
Cystatin C	>1.03 mg/L
Blood urea nitrogen (BUN)	>20 mg/dL
**Inflammation System**	
C-reactive protein (CRP)	>3 mg/L

Supplementary Table S2. Competing risk analysis of the association between allostatic load burden and new-onset arthritis

Variables	Model1		Model2		Model3	
	sHR (95%CI)	*P*	sHR (95%CI)	*P*	sHR (95%CI)	*P*
AL						
Low burden	1.00 (Reference)		1.00 (Reference)		1.00 (Reference)	
Medium burden	1.05 (0.97 ~ 1.14)	0.200	1.06 (0.98 ~ 1.15)	0.160	1.08 (1.00 ~ 1.18)	0.064
High burden	1.06 (0.94 ~ 1.20)	0.330	1.06 (0.94 ~ 1.20)	0.310	1.11 (0.98 ~ 1.26)	0.099

AL: allostatic load; sHR: subdistribution hazard ratio; CI: confidence interval.

Death before the occurrence of new-onset arthritis was treated as a competing event.

Model1: Crude

Model2: Adjust depressive symptoms

Model3: Adjust depressive symptoms, age, sex, place of residence, education level, marital status, smoking status, drinking status, diabetes, hypertension, and dyslipidemia

Supplementary Table S3. Competing risk analysis of the association between depressive symptoms and new-onset arthritis

Variables	Model1		Model2		Model3	
	sHR (95%CI)	*P*	sHR (95%CI)	*P*	sHR (95%CI)	*P*
Depressive symptoms						
No	1.00 (Reference)		1.00 (Reference)		1.00 (Reference)	
Yes	1.60 (1.48 ~ 1.73)	**<0.001**	1.60 (1.48 ~ 1.73)	**<0.001**	1.50 (1.38 ~ 1.62)	**<0.001**

AL: allostatic load; sHR: subdistribution hazard ratio; CI: confidence interval.

Death before the occurrence of new-onset arthritis was treated as a competing event.

Model1: Crude

Model2: Adjust Allostatic load

Model3: Adjust Allostatic load，age, sex, place of residence, education level, marital status, smoking status, drinking status, diabetes, hypertension, and dyslipidemia

Supplementary Table S4. Sensitivity analyses of the associations of allostatic load and depressive symptoms with new-onset arthritis after additional adjustment for physical activity (*n*=3780)

Variables	Model1		Model2		Model3	
	HR (95%CI)	*P*	HR (95%CI)	*P*	HR (95%CI)	*P*
**AL**						
Low burden	1.00 (Reference)		1.00 (Reference)		1.00 (Reference)	
Medium burden	1.04 (0.92 ~ 1.17)	0.580	1.02 (0.90 ~ 1.16)	0.706	1.03 (0.91 ~ 1.17)	0.618
High burden	1.18 (0.98 ~ 1.41)	0.075	1.18 (0.98 ~ 1.43)	0.089	1.19 (0.98 ~ 1.44)	0.072
**Deps**						
No	1.00 (Reference)		1.00 (Reference)		1.00 (Reference)	
Yes	1.70 (1.52 ~ 1.91)	**<.001**	1.59 (1.42 ~ 1.79)	**<.001**	1.60 (1.43 ~ 1.81)	**<.001**

AL: allostatic load; HR: hazard ratio; CI: confidence interval.

Model1: Crude

Model2: Adjusted for age, sex, place of residence, education level, marital status, smoking status, drinking status, diabetes, hypertension, and dyslipidemia; the AL analysis was additionally adjusted for depressive symptoms, and the depressive-symptom analysis was additionally adjusted for AL.

Model3: Additionally adjusted for the three physical activity indicators
